# Unleashing nanofabrication through thermomechanical nanomolding

**DOI:** 10.1126/sciadv.abi4567

**Published:** 2021-11-19

**Authors:** Naijia Liu, Guannan Liu, Arindam Raj, Sungwoo Sohn, Mayra Daniela Morales-Acosta, Jingbei Liu, Jan Schroers

**Affiliations:** 1 Department of Mechanical Engineering and Materials Science, Yale University, New Haven, CT 06511, USA.; 2 Institute of Materials Science, University of Connecticut, Storrs, CT 06269, USA.

## Abstract

Advancements in nanotechnology require the development of nanofabrication methods for a wide range of materials, length scales, and elemental distributions. Today’s nanofabrication methods are typically missing at least one demanded characteristic. Hence, a general method enabling versatile nanofabrication remains elusive. Here, we show that, when revealing and using the underlying mechanisms of thermomechanical nanomolding, a highly versatile nanofabrication toolbox is the result. Specifically, we reveal interface diffusion and dislocation slip as the controlling mechanisms and use their transition to control, combine, and predict the ability to fabricate general materials, material combinations, and length scales. Designing specific elemental distributions is based on the relative diffusivities, the transition temperature, and the distribution of the materials in the feedstock. The mechanistic origins of thermomechanical nanomolding and their homologous temperature-dependent transition suggest a versatile toolbox capable of combining many materials in nanostructures and potentially producing any material in moldable shapes on the nanoscale.

## Teaser

Thermomechanical nanomolding puts a wide range of materials combinations in reach for nanofabrication.

## INTRODUCTION

The ever-increasing demands from applications of nanodevices require advancements in nanofabrication methods. Ideally, a fabrication method can fabricate a range of materials, length scales, shape features, and a controlled elemental distribution within the nanostructure. This demand spans across broad application fields (*1*, *2*) from optics (*3*–*5*), electronics (*6*–*8*), life science (*9*, *10*), and energy harvesting (*11*, *12*) to quantum science (*13*). To realize such applications, a wide range of methods have been developed (*1*, *4*, *13*, *14*). However, most of today’s nanofabrication methods are limited in specific aspects (*2*). For example, vapor-liquid-solid growth, as an established method for high-quality nanowires and even heterostructures, is limited to material choices that generally exclude metals (*15*, *16*). On the other side of the spectrum is the chemical synthesis of colloidal nanoparticles. Highly scalable for mass production of many materials, it lacks versatility in the control over shape, dimension, and pattern (*17*, *18*). Although many of these methods find specific applications, a more versatile nanofabrication toolbox is required to address the rising demand for advanced nanotechnology, particularly for those where combinations of materials and length scales are required.

To develop a nanofabrication method that allows control over size, shape, chemistry, and the distribution of elements within the nanowire to result in material combinations requires a thorough understanding of the underlying fabrication mechanisms controlling length, composition, and material transport. Recently, thermomechanical nanomolding (TMNM) has been realized in metals and ordered phases, proposing a new molding principle that can be explored for nanofabrication (*19*–*21*). Although some aspects of the underlying mechanistic of TMNM have been suggested previously, an overall understanding of TMNM and its temperature and size scaling remains elusive (*21*, *22*). In this work, we identify the size- and temperature-dependent underlying mechanism of TMNM. Specifically, we reveal that the high-temperature deformation mechanism is diffusion dominated and occurs on the material-mold interface. With decreasing temperature or increasing size, a transition to dislocation slip–controlled growth occurs. This transition, which is material dependent, is used here to develop a versatile nanofabrication toolbox, offering the ability to fabricate a wide range of length scales, length scale combinations, materials and material combinations, and elemental distributions that include fabrication of heterostructures of a wide range of materials (*23*, *24*) in a high-throughput manner (*25*, *26*).

## RESULTS

### Underlying mechanisms of TMNM

**Fig. 1.**
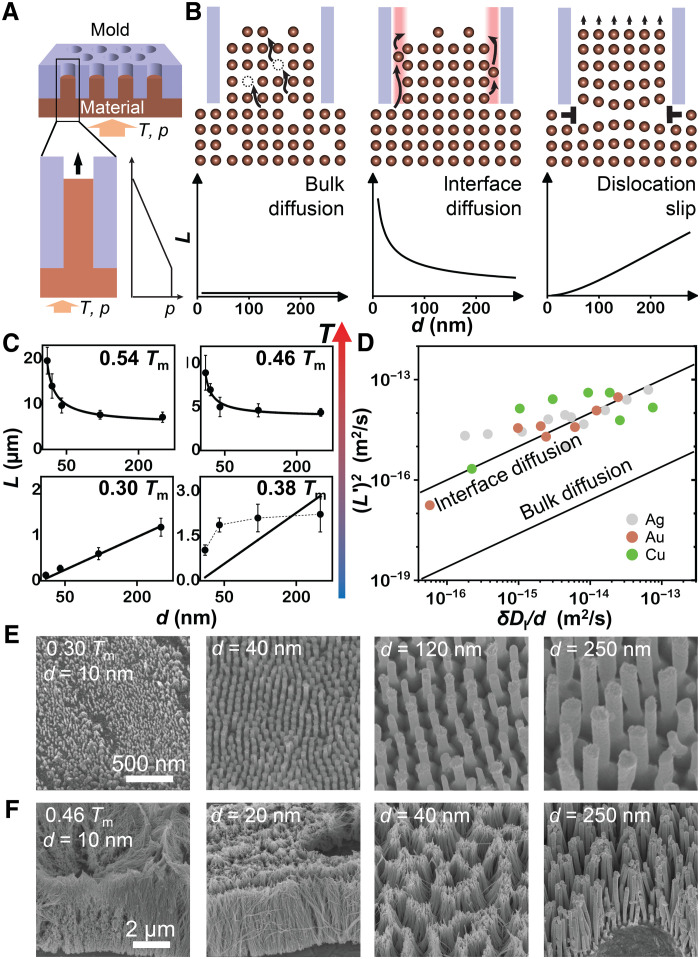
Mechanism of TMNM. (**A**) TMNM uses temperature and mechanical pressure to mold feedstock material into nanomold arrays. (**B**) Discussed material transport mechanisms on this length scale result in different length scaling, *L* versus *d*. Bulk diffusion (Eq. 1) results in *L*(*d*) ∝ const, interface diffusion gives $L(d)\propto \frac{1}{\sqrt{d}}$ (Eq. 2), and, for a dislocation slip mechanism, *L*(*d*) ∝*d^x^* (*x* ∈ [1,2]) (Eq. 3). (**C**) *L*(*d*) scaling experiments reveal the temperature-dependent mechanism for TMNM of Ag. Interface diffusion dominates TMNM at high temperatures, *T* > 0.4 *T*_m_, whereas dislocation slip takes over at low temperatures, *T* < 0.4 *T*_m_. (**D**) To compare TMNM across different systems, we normalize the forming length *L* to $L\prime =L/\sqrt{\frac{8p\Omega t}{k_{B}T}}$. Absolute values of experimentally determined *L* from Au, Ag, and Cu suggest an interface diffusion mechanism. The superimposed lines represent the magnitude of normalized molding length for interface diffusion, (*L*′)^2^ = δ*D*_I_/*d* and bulk diffusion, (*L*′)^2^~*D*_L_/4 (section S3). (**E** and **F**) Images of Ag nanowires corresponding to the data in (C).

TMNM fabricates nanostructures by driving feedstock material under an applied pressure and typically elevated temperature into a nanopatterned hard mold (Fig. 1A). Potential underlying mechanisms that control this process are bulk diffusion, interface diffusion, and dislocation slip (Fig. 1B) (*20*, *22*, *27*). To identify the mechanisms underlying TMNM, we analyzed the scaling relations of molding length (*L*) versus molding conditions. *L*(*d*) (with all other molding conditions fixed) is well distinguishable between different potential mechanisms and hence allows to identify the underlying mechanism (Fig. 1B).

Scaling for bulk and interface diffusion is based on Fick’s law, where material transports down a chemical potential gradient that is generated by the molding pressure and results in (section S1)

$$L=\sqrt{{L_{0}}^{2}+\frac{2p\Omega t}{k_{B}T}D_{L}}$$ (1)

for bulk diffusion and

$$L=\sqrt{{L_{0}}^{2}+\frac{8p\Omega t}{k_{B}T}(\delta D_{I}/d)}$$ (2)

for interface diffusion, where *D*_L_ and *D*_I_ are bulk and interface diffusivity of the feedstock material, δ is the thickness of the interface layer, Ω is the average atomic volume, *L*_0_ is an integration constant (section S1), *T* is the molding temperature, *t* is the molding time, and *p* is the hydrostatic pressure in the feedstock. *p* is controlled through the uniaxial compressive pressure on the surface of the feedstock and can be as low as one-third of the uniaxial pressure (sections S1 and S4). *L*(*d*) scaling for bulk diffusion is *L*(*d*) = const, and $L(d)\propto \frac{1}{\sqrt{d}}$ for interface diffusion (Fig. 1B). Because interface diffusivity is four to six orders of magnitude higher than bulk diffusivity for most metals (*D*_I_ ~*D*_L_ × 10^4 − 6^) (*28*–*30*), *L* based on interface diffusion into nanosize molds is predicted here to be much larger than that of bulk diffusion under similar molding conditions. Dislocation slip sets in when applied pressure exceeds the resistance induced by the shear strength (τ_I_) of the nanowire-mold interface. The scaling for dislocation slip mechanism is based on dislocation-mediated deformation, which pushes a nanowire to slip into the mold cavity (section S1). This process results in

$$L=\frac{\mathrm{pd}}{4\tau_{I}}(1-e^{-\alpha {b\tau }_{I}u\rho \mathrm{dt}})$$ (3)

where *b* is the magnitude of Burgers vector, *u* is the average propagation velocity of a dislocation, ρ is the density of dislocations in the steady deformation state of the feedstock, and α is a constant. Under typical molding conditions, the dislocation slip mechanism gives *L*(*d*) ∝*d^x^* (*x* ∈ [1,2])(Fig. 1B, fig. S2, and section S2).

We will use the distinct scaling behaviors for *L*(*d*) shown in Eqs. 1 to 3 to determine, experimentally, through scaling experiments the underlying mechanisms for TMNM for a given set of processing parameters (Fig. 1, C and D). Scaling experiments with Ag at various homologous temperatures (*T*_h_) reveal that the underlying mechanism changes from interface diffusion ($L(d)\propto \frac{1}{\sqrt{d}}$) at high *T*_h_ of 0.54 and 0.46 *T*_m_ to a dislocation slip mechanism (*L*(*d*) ∝ *d^x^*) at low *T*_h_ of 0.30 *T*_m_ (Fig. 1, C, E, and F). When considering a range of metals including Au, Cu, and Ag, the absolute values for *L* molded at high *T*_h_ match well with the magnitude predicted by the interface-diffusion mechanism, which is about three orders of magnitude higher than bulk diffusion (Fig. 1D and sections S3 and S5). These scaling experiments reveal diffusion-dominated TMNM at high *T*_h_ > 0.4, and the diffusion occurs on the interface. At low *T*_h_ < 0.4, dislocation slip mechanism dominates. This transiting mechanism also has similarities to the transition from dislocation-dominated power law creep to diffusion-controlled creep (Coble creep) within classical creep models (*31*). At temperatures close to the transition temperature of *T*_h_ ~ 0.4, the two mechanisms occur simultaneously (*32*–*35*). This can be concluded from the experimental finding that the *L*(*d*) scaling differs from the prediction of either diffusion or dislocation mechanism and can be better described by a superposition of both mechanisms (Fig. 1C).

### Range of length scales and materials

**Fig. 2.**
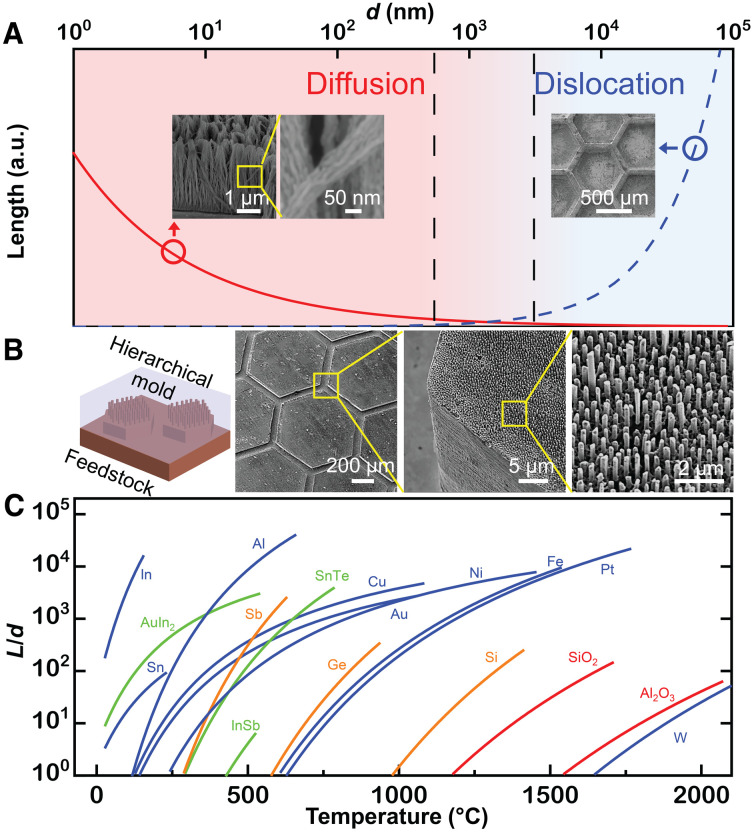
Materials and length scales that can be realized through TMNM. (**A**) Estimated (Eqs. 2 and 3) molding length as a function of molding dimension at intermediate molding temperature shows the transition of the dominating molding mechanism in TMNM from interface diffusion controlled to dislocation slip. TMNM can fabricate a large range of length scales from 5 nm (Ag, *L* ~ 8 μm) controlled by diffusion to millimeters (Au, ~1 mm) by dislocation. (**B**) Au sample hierarchical structures composing of a hexagonal micropattern (1 mm, through dislocation slip) combined with nanowire arrays (250 nm, through interface diffusion). (**C**) Calculated molding aspect ratio (*L*/*d*) according to Eq. 2 for interface diffusion as a function of temperature for representative materials from metals (blue), nonmetals (orange), oxides/ceramics (red), and ordered phases (green) including various functional materials (section S6 and table S1).

A transition in mechanism controlling TMNM does occur not only with temperature but also with molding size. For a given temperature, the scaling *L*(*d*) ∝ *d^x^* for dislocation slip– and $L(d)\propto \frac{1}{\sqrt{d}}$ for interface diffusion–dominated growth causes the controlling mechanism of TMNM to change from interface diffusion-controlled for small *d* to dislocation slip for large *d* (Fig. 2A). The transition in mechanism enables molding over a wide range of length scales from a single-digit nanometer to the millimeter scale (Fig. 2A). TMNM can readily fabricate ultrathin nanowires down to 5 nm in diameter through diffusion, with an aspect ratio exceeding 1000. This lower boundary is currently set by the nanomolds and likely not by the limitations of TMNM. It is challenging to fabricate molds with smaller diameter. Wires of diameter > 1 μm can be readily fabricated through a dislocation slip–dominated TMNM. This broad formability range also suggests possibilities for the fabrication of hierarchical structures through the combination of the two mechanisms (Fig. 2B). A mold composing of nano- and microfeatures can be replicated through a one-step molding operation. Here, for the same processing temperature, the micro features are replicated through a dislocation slip–dominated mechanism and the nanofeatures through an interface diffusion–controlled mechanism, leading to the moldability of complex hierarchical nano-micro patterns.

One important measure of the versatility of a nanofabrication method is the range of materials that can be fabricated. For interface diffusion–dominated growth, nanofabrication ability can be estimated from the temperature dependence of the diffusivity. Assuming typical conditions for TMNM, one can estimate nanomoldability by calculating the aspect ratio (*L*/*d*) that can be formed through Eq. 2. Specifically, we assume *p* = 1000 MPa, *t* = 10 min, and *d* = 40 nm and map the moldability for representative materials from pure metals, nonmetal elements, oxides and ceramics, and ordered phases (Fig. 2C). Using temperature-dependent diffusivity data from the literatures, Eq. 2 under the specific conditions suggest that a wide range of materials can be fabricated using TMNM. These materials even include W, SiO_2_, and Al_2_O_3_. Taking practicality into account, the processing temperature is an important aspect. In general, processing becomes increasingly challenging with increasing required processing temperature. This is due to reactions with the mold or the processing environment. Further, when considering TMNM as a processing step during device fabrication, the temperature compatibility with other materials and processes is another limiting factor. For example, if using silicon in conjunction in a device or as a mold for TMNM, then temperatures are limited to less than ~700°C. This would still allow to TMNM most metals with a *T*_m_ < 2100°C including Pt, Fe, and Ni, as well as various nonmetals, for example, Ge and Sb and most ordered phases. If TMNM is used in conjunction with polymers, then temperatures are limited to less than ~300°C. Here, metals with *T*_m_ < 1100°C such as Au, Cu, and Al can be used and even some functional materials including SnTe and InSb. When combining TMNM with biomaterials, temperatures are limited to ~100°C. Hence, only metals with *T*_m_ < 600°C such as Bi, In, and Sn can be considered.

### Controllable fabrication of heterostructures

**Fig. 3.**
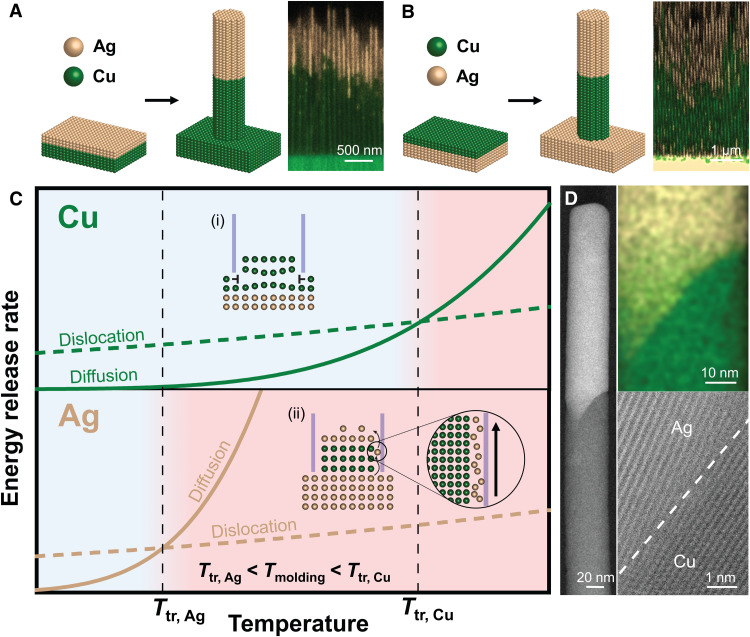
TMNM using multilayer feedstock. (**A** and **B**) Heterostructure nanowires are fabricated when using layers as feedstock. Here, we use Ag/Cu layers as example. The fabricated heterostructures are with distinct regions of essentially pure Ag and Cu. When using Ag/Cu-layered structure with Ag layer facing the mold and Cu away from the mold, the order in the heterostructure nanowires is identical to the order in the feedstock (A). When using a Cu/Ag-layered structure with Cu layer facing the mold and Ag away from the mold, however, the order in the heterostructure nanowires (Ag─Cu) has reversed over that of the feedstock Cu/Ag (B). (**C**) Temperature-dependent molding mechanisms for Ag and Cu where the transition temperature (*T*_tr_) is shown, which indicates the transition from dislocation slip–dominated to an interface diffusion–dominated molding mechanism. In the case of (A) and (B), the higher interface diffusivity in Ag results in lower *T*_tr_ than Cu. A molding temperature of *T*_tr, Ag_ < *T*_molding_ < *T*_tr, Cu_ results in the formation of Cu nanowire by dislocation slip, where the Ag atoms surpass on the mold/nanowire interface of the Cu region through the interface diffusion process. (**D**) Scanning transmission electron microscopy characterization reveals an atomically sharp Ag─Cu interface that forms during this process.

The strong absolute temperature dependence and strong homologous temperature dependence of the diffusivity that also causes a transition of the underlying mechanism controlling TMNM further offer the possibility for the control of elemental distributions to fabricate a wide range of heterostructure nanowires. This possibility can be further expanded by using, instead of homogeneous feedstock, layered combinations of materials as feedstock (Fig. 3). Heterostructure nanowires are of particular interests for many applications. Examples include nanodevices where the operating principles rely on functional interfaces, such as photodetectors (*36*), field-effect transistors (*26*, *37*), and light-emitting diodes (*38*).

To demonstrate how heterostructure nanowires can be fabricated through TMNM, we use Cu and Ag layers and consider different orders of these layers in the feedstock (Fig. 3 and fig. S5). When layering Ag above Cu and forming at 300°C, we obtain nanowires with clear separation of Ag and Cu that are arranged in the same order than in the feedstock (Fig. 3A and fig. S6). However, when changing the order in the feedstock material, Cu in front of Ag, TMNM reverses the order in the heterostructure nanowire. Ag surpasses the Cu and forms the tip of the nanowire followed by Cu (Fig. 3B and fig. S7). This change in the order of the feedstock and the heterostructure nanowire originates from the different underlying molding mechanisms occurring in Ag and Cu at the same absolute temperature. Whereas Ag forms via interface diffusion, Cu forms through dislocation slip at the molding temperature of 300°C. The reason for this behavior is that both mechanisms have element- and processing condition–specific temperature regions over which they dominate. To appreciate the existence of a material- and processing-specific transition temperature, *T*_tr_, one has to consider that, within TMNM, the driving force for nanowire growth is to release the pressure gradient (Fig. 1A). The dominating mechanism is the one with the fastest energy release rate, which is approximately reflected in the fastest growth rate (d*L*/d*t*) (Fig. 3C). Under certain molding conditions, the growth rate within an interface diffusion–controlled mechanism is defined by the interface diffusivity, d*L*_I_/d*t* ∝ *D*_I_, and within a dislocation slip–controlled mechanism by the dislocation density, ρ, and the average propagation velocity of the dislocations, *u*, giving d*L*_d_/d*t* ∝ *u*ρ. With increasing temperature, *D*_I_ increases exponentially, much faster than the increase of *u*ρ with temperature (*39*). Thus, the dominating mechanism changes from dislocation slip to interface diffusion controlled at *T*_tr_, which is defined by d*L*_I_/d*t* = d*L*_d_/d*t* (Fig. 3C). Under given molding conditions (*p* and *d*), *T*_tr_ is material specific and depends on the ratio of *D*_I_ and *u*ρ. A material can be expected to exhibit a high *T*_tr_ if its *D*_I_ is low. In addition, a high melting temperature also indicates high *T*_tr_ as the homologous temperature is low for the same absolute temperature. In the case of Ag─Cu heterostructures, the three times higher *D*_I_ for Ag compared to copper and the lower melting point of Ag result in a lower *T*_tr_ than that for Cu (*28*, *29*). Following the same line of thought, one can also expect a low *T*_tr_ for brittle materials (e.g., Ge and some intermetallics), where dislocation slip is essentially absent (low *u*ρ), while, for metals, *T*_tr_ is typically high.

To estimate *T*_tr_ of a material under specific conditions is generally challenging. However, for metals, our results suggest a *T*_tr_ ~ 0.4 to 0.5 *T*_m_. For Ag, this estimation suggests a *T*_tr_ ~ 200°C, lower than that of Cu of *T*_tr_ ~ 400°C (fig. S8). Therefore, at the molding temperature of 300°C, Cu molds by dislocation slip (*T* < *T*_tr, Cu_) and Ag by interface diffusion (*T* > *T*_tr, Ag_). Thereby, Ag atoms pass the formed Cu nanowire through the metal/mold interface and form Ag nanowire in front of Cu (Fig. 3C). When using Ag/Cu layered structure with Ag in front of Cu (Fig. 3A), Ag─Cu heterostructure nanowires form with distinct regions of essentially pure Ag and Cu. The order in the heterostructured nanowire is identical to the order in the feedstock as the dislocation mechanism present in Cu follows the growth of Ag.

Typically, diffusion-dominated TMNM forms nanowires of single-crystal structures with a defined orientation (<110> for face-centered cubic metals). On the other hand, nanowires formed through a dislocation slip process are polycrystalline or form a “bamboo” grain structure. We further investigated the Cu─Ag heterostructures and particularly the interface Ag/Cu with transmission electron microscopy (TEM). Scanning TEM reveals a sharp and clean separation between the Ag and Cu, part of the heterostructure with very similar yet a small difference in orientation (Fig. 3D and fig. S9). We speculate that the origin of an orientation misfit stems from different molding mechanisms of the Ag and Cu layer. In addition, further research is needed to explain this, especially the similar orientation between two layers.

## DISCUSSION

**Fig. 4.**
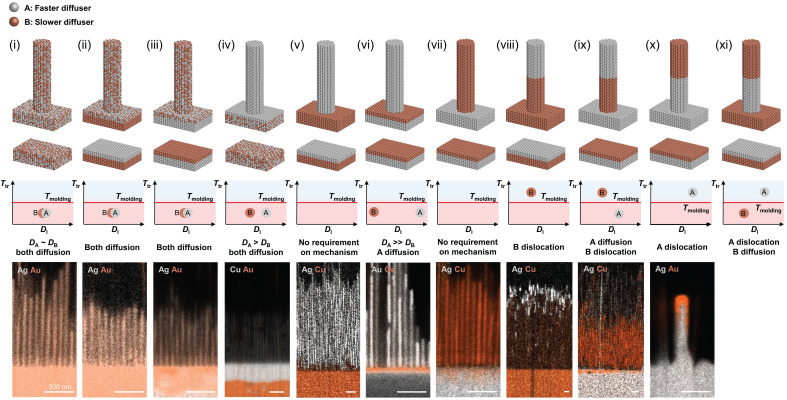
TMNM as a toolbox to control elemental distributions. The range of elemental distributions that can be achieved through TMNM using alloys or layered structure as feedstock. By using feedstock with different material combinations and considering their relative diffusivities and controlling molding mechanisms (molding above or below *T*_tr_) of each component, we can control the chemistry and structure of the nanowires. In the 11 cases listed, homogeneous alloys and layered element feedstock are used. Their relative diffusivities and *T*_tr_ for the involved elements relative to the molding temperature define the elemental distribution within the nanowire. This can be a homogeneous alloy (i to iii), single element (iv to vii), or heterostructure nanowires (viii to xi). The bottom row shows example systems for the specific cases. Further details of each case can be found in section S7.

On the basis of such a mechanism (Fig. 3), TMNM offers the possibility to control the elemental distribution within the nanowire by designing the processing and material properties, and versatile nanostructures can be achieved (Fig. 4). One tool is the feedstock material where we consider alloys and layered structures. Further, the relative diffusivities of the involved elements (*D*_A_ and *D*_B_) have to be considered as they define the diffusion velocity of the elements, which defines the movement of each element in the feedstock. Which element-specific mechanism takes place is determined by the element-specific *T*_tr_ relative to the molding temperature. For example, homogeneous alloy nanowire can be fabricated from alloy or layered feedstock [Au_50_Ag_50_ as an example in Fig. 4 (i to iii)]. To realize homogeneous alloy nanowire from alloy feedstock, the alloy constituents require a similar diffusivity and their *T*_tr_ must be below the molding temperature. Using elements with different diffusivities to realize homogeneous alloy nanowire requires the use of layered structure as feedstock, and their order in the feedstock is defined by their relative diffusivity. The ability to realize homogeneous alloy nanowires from such range of feedstock is potentially powerful. For example, it suggests itself to be used to realize homogeneous alloy nanowires with elements that are difficult or unwilling, under equilibrium thermodynamic conditions, to form feedstock alloy. If, for example, A and B thermodynamically do not mix in their solid state (under equilibrium conditions), then they can still be mixed in the nanowire during TMNM as their formation and distribution within the nanowire are kinetically controlled, in the here-discussed case, by the relative diffusivities of the constituent elements.

TMNM can also fabricate single-element nanowires with various nanowire-substrate combinations (Fig. 4, iv to vii). Desired nanowire and substrate can be achieved using layered feedstocks with controlled layer sequence (Fig. 4, v to viii). This offers the ability to design the material combination (metal nanowires on semiconductor substrate; Fig. 4, vi) and metal nanowires on metal substrate [Fig. 4 (v and vii) as examples] in fabricated nanostructures, an important attribute for functional applications and device fabrication. One can also form a single-element nanowire using a homogeneous alloy feedstock and drive out solely the faster diffuser [pure Cu nanowire formed from Au_50_Cu_50_ as an example in Fig. 4 (iv)]. This requires that the element that is desired in the nanowire to have a higher diffusivity than the other component elements, and the molding temperature is higher than *T*_tr_ of all the components in the feedstock. This tool can, for example, be used to fabricate ultrapure nanomaterials by removing slow-diffusing impurities through TMNM.

Using layered structures as feedstock, TMNM can also realize heterostructure nanowires (Fig. 4, viii to xi). This requires that one of the elements molding is based on dislocation slip (*T* < *T*_tr_). The element for which the deformation mechanism is based on dislocation can either be in front of the layer of the element that deforms through diffusion (*T* > *T*_tr_) and surpassed by that layer or the section within the nanowire or be positioned behind the other layer, which can be based on either diffusion or dislocation, and maintains that position during TMNM. This process enables full control over the material combination and elemental distributions over heterostructure nanowires and will be powerful for advanced nanodevices fabrication with functional interfaces.

In conclusion, we revealed the underlying mechanism controlling TMNM, which undergoes a temperature- and size-dependent transition. At high temperature and small size, TMNM is controlled by diffusion occurring on the interface between the material and the mold. At low temperature and large size, the dominant mechanism switches to dislocation slip. This transition in mechanism is used here to control TMNM with a wide range of materials, length scales, and length scale combinations. By applying TMNM to feedstocks with material combinations, we can design the molding conditions to gain control over elemental distribution in the nanowires including homo- and heterostructures. As the underlying mechanisms of TMNM and their dependence on temperature and size are present in essentially all solid materials, TMNM suggests a paradigm shift for nano-applications from the previous approach of using what is available on the nanoscale to using what is desired.

## MATERIALS AND METHODS

### Preparation of feedstocks

For elemental metal feedstocks, high-purity raw materials (Au, Ag, and Cu, 99.99+% in purity) were melted and quenched into 3-mm rods. Alloy feedstocks (Au_50_Ag_50_ and Au_50_Cu_50_) were alloyed by arc-melting with high-purity constituent (99.99+% in purity), quenched into 3-mm diameter rods, and annealed at 500°C in argon for 10 hours to get a good mix. Rods of elemental and alloy feedstocks were then sliced into ~0.8-mm-thick discs and double-side polished for TMNM. For feedstocks with a layered structure, we used the prepared elemental feedstocks and deposited the top layer with magnetron sputtering.

### Thermomechanical nanomolding

We used commercially available anode aluminum oxide (AAO) templates (from InRedox) as hard mold in this work. The pore diameter (*d*) of these molds ranges from 250 to 5 nm. In TMNM, prepared feedstocks were attached against the hard mold, heated up to processing temperature (*T*) by Instron testing system equipped with heating plates, quickly loaded to loading pressure in 5 s, and then held for a certain time (*t*). Upon molding, AAO templates can be removed (demolding) by etching in 20 weight % (wt %) KOH or 10 wt % phosphoric acid at room temperature for 10 hours to get free-standing nanowire arrays.

### Characterization of nanowires

We characterized length, chemical, and structural information of fabricated nanowires with scanning electron microscopy (SEM) and TEM. The length of scaling experiments was measured in SEM upon demolded samples. To reveal the chemical information of heterostructure nanowires, we first cut a cross section of the material-mold combination upon molding and polished the cross section with an ion mill (Hitachi IM4000 system). These cross sections that contain information of both substrates and nanowires were then mapped by energy-dispersive x-ray spectroscopy (EDS) (BRUKER XFlash 5060FQ Annular EDS detector) in SEM (Hitachi SU8230 UHR Cold Field Emission SEM). For structural characterization of nanowires, material-mold combination upon molding was cut along growth direction and thinned down to ~60 nm with a focused ion beam (Helios G4 UX DualBeam system). We further revealed the atomic and the elemental distribution of nanowires by TEM (FEI Titan Themis).

## Supplementary Materials

### This PDF file includes:

Supplementary Text Figs. S1 to S12 Table S1 References
