# Supplementary material for: Unleashing nanofabrication through thermomechanical nanomolding
Source: Sci Adv. 2021 Nov 19;7(47):eabi4567. doi: 10.1126/sciadv.abi4567 (PMC8604398; doi:10.1126/sciadv.abi4567)
Supplement: Supplementary file 1 — Supplementary Text Figs. S1 to S12 Table S1 References [file sciadv.abi4567_sm.pdf]

Science Advances
AAAS

Supplementary Materials for

Unleashing nanofabrication through thermomechanical nanomolding

Naijia Liu, Guannan Liu, Arindam Raj, Sungwoo Sohn, Mayra Daniela Morales Acosta, Jingbei Liu, Jan Schroers*

*Corresponding author. Email: jan.schroers@yale.edu

Published 19 November 2021, *Sci. Adv.* **7**, eabi4567 (2021)
DOI: 10.1126/sciadv.abi4567

This PDF file includes:

Supplementary Text
Figs. S1 to S12
Table S1
References

Supplementary Materials

Supplementary Text

Supplementary Section 1: Molding mechanisms in TMNM

There are three possible mechanisms enabling the growth of nanowires in TMNM, that are bulk diffusion, interface diffusion, and dislocation slip mechanism. Each mechanism exhibits different scaling relations between molding length (*L*) and molding conditions including temperature (*T*), hydrostatic pressure (*p*), molding time (*t*), and mold diameter (*d*). This gives the possibility to determine the mechanism behind TMNM by revealing experimental scaling behaviors. In this section, the scaling laws in each mechanism will be derived.

Bulk diffusion and interface diffusion: We quantify the scaling behavior in diffusion-based TMNM by considering the volume change of nanowires (*dV/dt*) as the result of the atom diffusion flux (*J*) through the cross-section area of diffusion path (*S*) in each nanowire, with:

$$\frac{\mathrm{d}V}{\mathrm{d}t} = S \cdot J \tag{S1}$$

Here the volume flux *J* (in unit of m/s) follows Fick's law $J = \frac{D}{\mathrm{k_B}T}\nabla(p\Omega)$, with *D* as the diffusivity and *Ω* the mean atomic volume in the feedstock material. This volume flux can be translated into the atomic flux *J'* (the number of atoms crossing unit area per unit time) by $J = J'\Omega$. Substituting *J* in eq. S1 and considering $V = \frac{\pi d^2}{4} \cdot L$, we get the control equation of growth rate (*dL/dt*):

$$\frac{\mathrm{d}L}{\mathrm{d}t} = \frac{4SDp\Omega}{\pi d^2 L \mathrm{k_B}T} \tag{S2}$$

, by integrating which gives:

$$L = \sqrt{{L_0}^2 + \frac{8SDp\Omega t}{\pi d^2 \mathrm{k_B}T}} \tag{S3}$$

Here L_0 is an integration constant related to the loading process and the entry effect in TMNM. In the case of bulk diffusion, where diffusion coefficient is measured by lattice diffusivity ($D = D_L$) and diffusion happens across the whole volume of the nanowire ($S = \frac{\pi d^2}{4}$), we have:

$$L = \sqrt{{L_0}^2 + \frac{2D_\mathrm{L}p\Omega t}{\mathrm{k_B}T}} \tag{S4}$$

In the case of interface diffusion, where diffusion coefficient is measured by interface diffusivity ($D = D_I$) and diffusion is limited in a thin interface layer of a thickness *δ* between the nanowire and mold ($S = \pi d\delta$), we have:

$$L = \sqrt{{L_0}^2 + \frac{8D_\mathrm{I}p\Omega t}{\mathrm{k_B}T}\frac{\delta}{d}} \tag{S5}$$

Dislocation slip: Nanowires grow by dislocation sliding down into mold in dislocation slip mechanism. Since the moving speed of nanowires in these nanocavities is super slow, by ignoring all inertial forces we have:

$$p_\mathrm{e}\frac{\pi d^2}{4} = \tau_\mathrm{I}\pi dL \tag{S6}$$

, where p_e is the pressure at the entrance of the nanocavity and τ_I is the shear strength along the nanowire-mold interface. In the process of TMNM, the total molding pressure is consumed partially in the feedstock material and partially in the nanocavities to achieve a free surface (p = 0) at the tips of nanowires. This pressure distribution results in a decreasing gradient from the feedstock to all the way through the nanowire and gives $p_e \leq p$. In a growing nanowire, p_e increases with increasing L according to eq. S6 until $p_e = p$. This sets a maximum length in dislocation-based TMNM by:

$$L \leq L_m = pd/4\tau_I \quad \text{(S7)}$$

We consider the growth of nanowires in this dislocation slip case is based on dislocation moving in the feedstock material driven by the pressure drop $p - p_e$. The control equation of the growth rate can be written by:

$$\frac{dL}{dt} = Cbu(p - p_e)N_d = Cbup\left(1 - \frac{L}{L_m}\right)N_d \quad \text{(S8)}$$

, where C is a material related constant, b is the magnitude of the Burgers vector, u is the average propagating velocity of dislocations, N_d the number of dislocations contributing to nanowire growth. Integrating eq. S8 and considering $L_{(t=0)} = 0$ gives:

$$L = L_m\left(1 - e^{-\frac{CbuN_d pt}{L_m}}\right)$$

Substitute L_m with eq. S7, then we have:

$$L = \frac{pd}{4\tau_I}\left(1 - e^{-\alpha b\tau_I u\rho dt}\right) \quad \text{(S9)}$$

, where $\alpha = C\pi$ is a constant, and $\rho = N_d/\frac{\pi d^2}{4}$ is the dislocation density in the feedstock at steady deformation state.

Supplementary Section 2: Scaling results from the theoretical models

Equation S4, S5 and S9 represent the scaling law of the three mechanisms of bulk diffusion, interface diffusion, and dislocation slip. To simplify the discussion, we assume $L_{(t=0)} = 0$. By using the same molding material and keeping T, t, p to be constant, three mechanisms result in very different scaling of L vs. d, that can be readily revealed by experiments. In the case of bulk diffusion, eq. S4 gives

$$L \propto d^0$$

, while in interface diffusion eq. S5 shows

$$L \propto d^{-1/2}$$

This means a constant length in bulk diffusion and a decreasing length in interface diffusion dominated cases when molding diameter is increasing.

In the case of dislocation slip mechanism, we need more discussion to simplify the format of eq. S9. Consider a format of $L = md(1 - e^{-kd})$ where $m = \frac{pd}{4\tau_I}$ and $k = \alpha b\tau_I u\rho t$ are constants resulted from eq. S9, the derivative of eq. S9 gives $(L(d))' = m(1 - e^{-kd}) + mkde^{-kd} > 0$ and $(L_{(d=0)})' = 0$ under practical molding conditions, which gives an increasing L with d. To get an

intuitionistic idea of the scaling behavior in eq. S9, we approximate it with a power function $L = \mathrm{n}d^{x}$, which is more comparable to the cases in bulk diffusion ($L \propto d^{0}$) and interface diffusion ($L \propto d^{-1/2}$), and reveal the range of x that can fit eq. S9 well. Considering this power function is equivalent to a linear format $\ln L = \ln n + x(\ln d)$, the approximation above results in a linear fitting to eq. S9 in format: $\ln L = \ln \mathrm{m} + (\ln d) + \ln(1 - \mathrm{e}^{-\mathrm{k}d})$, where we have $\frac{\mathrm{d}(\ln L)}{\mathrm{d}\,(\ln d)} = 1 + \frac{\mathrm{k}\mathrm{e}^{-\mathrm{k}d}}{1-\mathrm{e}^{-\mathrm{k}d}}\frac{\mathrm{d}\,(d)}{\mathrm{d}\,(\ln d)} = 1 + \frac{\mathrm{k}d}{\mathrm{e}^{\mathrm{k}d}-1}$. This derivative is a decreasing function with *d* for $d \in [0, +\infty)$, when $d = 0$, $\frac{\mathrm{d}(\ln L)}{\mathrm{d}\,(\ln d)} = 2$, and when $d \to +\infty$, $\frac{\mathrm{d}(\ln L)}{\mathrm{d}\,(\ln d)} = 1$. Thus, $1 \le \frac{\mathrm{d}(\ln L)}{\mathrm{d}\,(\ln d)} \le 2$ results in a linear fitting with slope $1 \le x \le 2$. And we achieve an approximation of eq. S9 with

$$L \propto d^{x}, x \in [1, 2]$$

A detailed numerical result of $L(d)$ with varies constants *m* and *k* is plotted in Fig. S2A, where by tunning these constants the curve eq. S9 is shifting between first and second order power functions. In practical molding conditions of loading pressure ~ 500 MPa and τ_{I} ~ 10 MPa, we have *m* ~ 10 that agrees with the fitting results of experimental data (Fig. 1C and Fig. S2B). However, the estimation of *k* remains difficult as there is an unknown constant α in its expression.

When using scaling experiments as a tool to reveal the underlying mechanism, we are aiming to distinguish between dislocation slip, interface diffusion or bulk diffusion-controlled growth. Within TMNM, $L = L(t, T, p, d)$. We choose $L(d)$ as it's scaling for bulk diffusion ($L(d)$ = const), interface diffusion ($L(d) \propto \frac{1}{\sqrt{d}}$), and dislocation slip ($L(d) \propto d^{x}$ $(x \in [1, 2])$) are well distinguishable. The other parameters, *t*, *T*, *p* yield the same scaling for *L*, hence do not allow to distinguish between bulk and interface diffusion. For example, the scaling of: $L(p) \propto \sqrt{p}$, $L(t) \propto \sqrt{t}$, and $L(T) \propto e^{-\mathrm{C}/T}$ is the same for bulk and interface diffusion dominated cases.

The scaling between *L* and *t* is also considered as a criterion of dislocation-based from diffusion-based mechanisms. In the case of diffusion-based mechanisms, eq. S4 and eq. S5 give an increasing L with time with $L \propto t^{1/2}$. In the case of dislocation-based mechanism, on the other hand, eq. S9 sets a limitation of *L* and results in a shotting up of length then a quasi-constant state after a short time. This is revealed both numerically and experimentally in Fig. S3.

Supplementary Section 3: Absolute value analysis between bulk and interface diffusion

Besides the scaling behaviors, we also reveal the huge difference in absolute length values expected by bulk and interface diffusion mechanisms, with results summarized in Fig. 1D.

To compare data measured with varies molding conditions (*T*, *p*, and *t*), we first calculated the normalized molding length (*L'*) by $L' = \sqrt{\frac{L^2}{8p\Omega t/\mathrm{k_B}T}}$. In TMNM, *L'* will be totally decided by molding diameter and the diffusivity of molding material. Furthermore, in the case of bulk diffusion, with eq. S4 we have:

$$L_{\mathrm{B}}' = \sqrt{D_{\mathrm{L}}/4} \qquad \text{(S10-1)}$$

And in the case of interface diffusion, eq. S5 gives:

$$L_{\mathrm{I}}' = \sqrt{\delta D_{\mathrm{I}}/d} \qquad \text{(S10-2)}$$

In typical molding conditions, we consider $d \sim 100$ nm, $\delta \sim 1$ nm, and $D_I \sim 10^4 D_L$. One can derive from eq. S10-1 and eq. S10-2:

$$(L_I')^2 = \delta D_I/d \sim 400\ (L_B')^2 \tag{S11}$$

For a given molding material (δD_I) and molding diameter, the normalized molding length (L') under both diffusion-based mechanisms can be then estimated with eq. S11 and compared with experimental data as shown in Fig. 1D.

Supplementary Section 4: Uniaxial pressure, hydrostatic pressure, and pressure at the entrance

In the theoretical analysis of TMNM, we need to consider three different pressures, the uniaxial compressive pressure (the loading pressure defined by F/A, where F is the loading force and A is the surface area of the feedstock), the hydrostatic pressure inside the feedstock (p), and the pressure at the entrance of nanocavities (p_e). The pressure at the entrance of the nanocavities (p_e) does not always equal to the hydrostatic pressure (p) in the feedstock, which can be as low as one third of the uniaxial compressive pressure (F/A). In TMNM, p_e is not constant but can change with time and the length of the forming nanowires. For derivation of scaling equations (supplementary section 1), the relation between p_e and p is demonstrated in the dislocation dominated mechanism (eq. S6). For diffusion dominated cases, however, we approximate p_e to p to simplify the scaling behavior of $L(t, T, p, d)$ (eq. S1 to S5). $p_e = p$ is a reasonable approximation, but it deserves more mathematical discussion for the pressure and length evolution in TMNM.

In both cases, diffusion and dislocation, p_e is not constant but varies with the length of the nanowire. In TMNM, each nanowire experiences a force balance between the applied pressing force at the entrance ($p_e S_A$) and the resistance from the material-mold interface ($\tau_I \pi d L$):

$$p_e S_A - \tau_I \pi d L = \frac{\mathrm{d}(mv)}{\mathrm{d}t} = m\frac{\mathrm{d}v}{\mathrm{d}t} + v\frac{\mathrm{d}m}{\mathrm{d}t} \sim 0$$

, where S_A is the cross-section area of the nanowire, m is the mass of the nanowire, τ_I is the shear strength along the nanowire-mold interface, and v is the moving speed of the nanowire. This leads to:

$$\begin{cases} p_e = \frac{\tau_I \pi d L}{S_A} & \left(\frac{\tau_I \pi d L}{S_A} \le p\right) \\ p_e = p & \left(\frac{\tau_I \pi d L}{S_A} > p\right) \end{cases} \tag{S12}$$

In the dislocation case, the growth of the nanowire is driven by ($p - p_e$). When p_e increases with L and approaches the full value of hydrostatic pressure p in the feedstock, the growth rate of nanowire reduces to zero, setting a maximum length to TMNM of $L_m = pd/4\tau_I$ (eq. S7). The additional calculations we did for the variation of p_e and L vs. time for the dislocation case are plotted in Figure S10.

In the case of interface diffusion, the growth of the nanowire is driven by the pressure gradient that is defined by p_e/L. This process can be further divided into two steps: (i) when $L \le L_m = pd/4\tau_I$ ($t \le t_m$), $p_e = \frac{\tau_I \pi d L}{S_A}$. This gives the growth rate of the nanowire:

$$\frac{\mathrm{d}L}{\mathrm{d}t} \propto \frac{p_e}{L} = \frac{\tau_I \pi d}{S_A} \qquad (t \le t_m)$$

This constant growth rate results in a linear relation between L and t for interface diffusion-controlled growth.

(ii) when $t > t_m$, $p_e = p$, and the growth rate is defined by:

$$\frac{\mathrm{d}L}{\mathrm{d}t} \propto \frac{p_e}{L} = \frac{p}{L} \qquad (t > t_\mathrm{m})$$

In contrast to the case of dislocation slip controlled growth, nanowires will keep growing in the atomic diffusion-controlled region down this pressure gradient (Fig. S11). The integration of the equation above leads to a square root relation between L and t, which is shown in eq. (S5). Thus, the relation between L and t in (ii) can be fully represented by:

$$\begin{cases} L = \frac{p_e \Omega}{L k_B T} \frac{S}{S_A} D_I t = \frac{\tau_I \Omega}{k_B T} \frac{16\delta}{d^2} D_I t & (t \le t_\mathrm{m}) \\ L = \sqrt{{L_0}^2 + \frac{8p\Omega}{k_B T} \frac{\delta}{d} D_I t} & (t > t_\mathrm{m}) \end{cases} \qquad \text{(S13)}$$

, where L_0 is an integration constant, and S is the cross-section area of the interface layer. For cylindrical shaped nanowires, $S = \pi\delta d$, and $S/S_A = 4\delta/d$. The variation of p_e, p_e/L and L vs. time in interface diffusion case are plotted in Figure S11.

For diffusion based TMNM, the growth of nanowires is driven by fast atomic transport through interface diffusion. As a result, t_m is a very short time when compared to the experimental time scale of molding (~10 sec by estimation compared to typical experiment times ~300 sec). It is also important to mention that t_m is also much shorter for diffusion-controlled growth than for dislocation-controlled growth. Our experimentally determined L_m ~ 1μm when using typical molding diameters (Fig. S3 and S6).

Strictly speaking, eq. S1 to S5 are only valid for $t > t_m$. However, since t_m is very short compared to the experimental time scale, the potential difference in describing the $L(t, T, p, d)$ through eq. S1 to S5 can be expected to be small.

Supplementary Section 5: Size effect on the dominating diffusion mechanism

The activation energy for interface diffusion is much lower than that for bulk diffusion, leading to a decreasing difference between D_I and D_B with increasing temperature. In bulk materials D_I and D_B cross over at a temperature below the liquidus temperature which leads to a transition of deformation mechanism from grain boundary diffusion to lattice diffusion at high temperatures, such as in diffusional creep. For nanomaterials, as the case in TMNM, size effect also needs to be considered due to a drastic increase of volume portion of interface layers/grain boundary layers.

In TMNM, the ratio between the contribution of interface diffusion and bulk diffusion is:

$$\frac{\pi d \delta D_I}{\pi d^2 D_B/4} = \frac{4\delta D_{I\,0}}{d D_{B\,0}} e^{(Q_B - Q_I)/k_B T}$$

, where Q_I and Q_B are the activation energy of interface and bulk diffusion, resepectively. By defining T_c as the temperature where bulk and interface diffusion have the same contribution to the growth of nanowires,

$$\frac{\pi d \delta D_I}{\pi d^2 D_B/4} = 1$$

we have:

$$\frac{Q_B - Q_I}{k_B T_c} = \ln\left(\frac{dD_{B\,0}}{4\delta D_{I\,0}}\right)$$

Solving for T_c gives:

$$T_c = \frac{Q_B - Q_I}{k_B \ln\left(\frac{dD_{B\,0}}{4\delta D_{I\,0}}\right)} \tag{S14}$$

This equation reveals that T_c is a function of the molding diameter (or grain size) d. For bulk materials, it has been studied for diffusional creep that lattice diffusion can dominate over grain boundary diffusion at high temperature of $T_c \sim 0.8\ T_m$, but for nanocrystalline materials, T_c can increase beyond T_m with decreasing d, so that no transition from interface to bulk diffusion takes place. To show the size dependence of this transition, we calculated the function of T_c/T_m vs. d for some typical materials used in this study, Au, Ag, and Cu. The results are plotted in Figure S12.

This plot reveals that for typical length scales (e.g., d < 1 μm) and temperature range of thermomechanical nanomolding ($T < T_m$), the diffusion-controlled regime is always dominated by interface diffusion. For large molding dimensions beyond nanomolding (e.g., d > 10 μm), similar to bulk materials, bulk diffusion will dominate at high temperatures.

Supplementary Section 6: Moldability mapping of TMNM

In Fig 2C we plot the moldability of representative materials by predicting their molding aspect ratio (L/d) as a function of molding temperature. Based on the analysis of molding mechanisms in TMNM (eq. 2 and eq. S5), the moldability of materials is defined by their interface diffusivity,

$$L/d = \sqrt{\frac{8p\Omega t}{\mathrm{k_B}T}\frac{\delta}{d^3}D_I} \tag{S15}$$

eq. S15 predicts an expected aspect ratio in TMNM by assuming certain molding conditions (specifically we use d = 40 nm, a loading pressure of 1 GPa, t = 600 s, and assume δ = 1 nm and $\Omega = \frac{4}{3}\pi r^3$, where r is the atomic radius). However, with reported data of only a few materials, to find the interface diffusivity is generally challenging. Thus, instead of using interface diffusivity for all materials, we pick bulk diffusivity and enlarge them to reasonable magnitude when interface diffusivity is not available. Specifically, our data treatment follows the principles of:

(1) When interface diffusivity (or grain boundary diffusivity) data is available from literature, we use the interface diffusivity for calculating the aspect ratio of nanowires. These materials include: Au, Cu, and Ni.

(2) If there is no interface diffusivity data available from literatures, we use diffusivity data that are measured from fine-grained samples (lateral samples and thin films). With the existence of diffusion short-circuits (*40*) along their high volume ratio grain boundaries, fine-grained samples are of similar magnitude of diffusivity with interface diffusivity. These materials used include Sn and $AuIn_2$.

(3) If both interface diffusivity and diffusivity data from fine-grained sample are not available, we have to use self-diffusivity data from bulk samples instead. Generally, it is reported that for typical materials the interface diffusivity (or grain boundary diffusivity) is 4 to 6 orders of magnitudes higher than their bulk self-diffusivity at intermediate temperature where TMNM usually applies

(*28-30, 41-43*). So, in this case we apply a factor of 10^4 to $D_{B\,0}$ by estimating $D_I \sim 10^4 D_B$. Despite the possible difference of the activation energy Q (*41*), such an estimation presents reasonable length prediction in TMNM (*20*). These materials include In, Al, Fe, Pt, W (diffusivity fitted from reported experimental results), Sb, Ge, Si, InSb, SnTe, SiO_2, and Al_2O_3.

All data treated can be found in Table S1 with original references.

Supplementary Section 7: Details of cases in TMNM as a toolbox

There are 11 cases listed in Fig. 4 showing the controlled versatile fabrication in TMNM. Below we provide more details of each case. We use A and B to represent two components/layers in the feedstock, and assume A is the faster diffuser without further notice.

Case (i): When using AB solid solutions as feedstock and processing in a diffusion dominated region, one can fabricate nanowires with the same composition as feedstock if the components, A and B, are with similar diffusivities as each other. In this case, nanowires grow up by diffusion with atomic flux of A and B proportional to their composition in feedstock ($D_A \sim D_B$). One example of this case is TMNM of $Au_{50}Ag_{50}$ at 450°C and 40 nm, fabricated nanowires are with the same composition as feedstock. Here one should note that although Ag is a faster diffuser than Au as pure metals, Ag atoms have similar diffusivity as Au atoms in Au-Ag solid solution phases (*43*).

Case (ii): When using A-B layered feedstock (A at the top) and processing at temperature $T > T_{c,\,A}$ and $T > T_{c,\,B}$, one can fabricate nanowires of AB mixture that stand on a substrate of B. In this case, nanowires grow up by interface diffusion that can bring in atoms of A and B at the same time. One example of this case is TMNM of Ag-Au layered feedstock (200 nm Ag layer at the top) at 450°C and 40 nm, fabricated nanowires are Au-Ag solid solutions.

Case (iii): When using B-A layered feedstock (B at the top) and processing at temperature $T > T_{c,\,A}$ and $T > T_{c,\,B}$, one can fabricate nanowires of AB mixture that stand on a substrate of A. The mechanism behind this case is the same as case (ii). One example of this case is TMNM of Au-Ag layered feedstock (200 nm Au layer at the top) at 450°C and 40 nm, fabricated nanowires are Au-Ag solid solutions.

Case (iv): When using AB solid solutions as feedstock and processing in a diffusion dominated region, one can fabricate pure A nanowires that stand on AB solid solution substrates if $D_A > D_B$. In this case, the composition of nanowires are controlled by the kinetic process with competition between diffusion of A atoms and B atoms. Always under the same temperature and pressure gradient, the atomic flux of A and B is solely defined by their diffusivities. And the faster diffuser (A) has higher flux and can eventually change the composition of grown nanowires to pure A. One example of this case is TMNM of $Au_{50}Cu_{50}$ at 450°C and 40 nm, fabricated nanowires are pure Cu standing on Au-Cu substrate.

Case (v): When using A-B layered feedstock (A at the top), one can fabricate pure A nanowires standing on pure B substrate. There is no specific requirement on mechanisms in this case. The top layer (A) will always grow first and this case can be achieved upon controlling of molding time. One example of this case is TMNM of Ag-Cu layered feedstock (700 nm Ag at the top) at 300°C and 40 nm, fabricated nanowires are pure Ag standing on pure Cu.

Case (vi): When using B-A layered feedstock (B at the top) and processing at temperature $T > T_{c,\,A}$ and $T > T_{c,\,B}$, one can fabricate nanowires of A that stand on a B-A layered substrate if $D_A >> D_B$. In this case, B does not have high enough diffusivity to enable nanowire growth, while A

atoms can diffuse through the grain boundaries of B layer and grow into the mold and form pure A nanowires. Specifically, in TMNM, for feedstocks with low diffusivities (B in this case) dislocation slip can become the dominating mechanism and also enable nanowire growth. So, it is critical in this case to choose brittle materials for B that dislocation mechanism barely exists. One example is TMNM of Ge-Au layered feedstock (200 nm Ge at the top) at 300°C and 40 nm, fabricated nanowires are pure Au standing on Ge-Au layered feedstock.

Case (vii): When using B-A layered feedstock (B at the top), one can fabricate pure B nanowires standing on pure A substrate. There is no specific requirement on mechanisms in this case and the mechanism behind is the same as case (v). One example of this case is TMNM of Cu-Ag layered feedstock (500 nm Cu at the top) at 300°C and 40 nm, fabricated nanowires are pure Cu standing on pure Ag substrate.

Case (viii): When using A-B layered feedstock (A at the top) and processing at temperature $T < T_{c,\mathrm{B}}$, one can fabricate nanowires of A-B heterostructure that stand on a substrate of B. In this case, nanowires of A form first until all A atoms are consumed. Then, B nanowires grow by dislocation slip and push the formed A parts up. One example of this case is TMNM of Ag-Cu layered feedstock (200 nm Ag at the top) at 300°C and 80 nm, fabricated nanowires are Ag-Cu heterostructure standing on pure Cu substrate.

Case (ix): When using B-A layered feedstock (B at the top) and processing at temperature $T > T_{c,\mathrm{A}}$ and $T < T_{c,\mathrm{B}}$, one can fabricate nanowires of A-B heterostructure that stand on a substrate of A. In this case, B nanowires form first until all B atoms are consumed. Then, A atoms diffuse through the interface between B nanowires and mold, and build up nanowire on top of B by epitaxial growth. During the growth of A, the dislocation dominated mechanism prevents B atoms from mixing with the atomic flux of A and forming AB mixture nanowires. One example of this case is TMNM of Cu-Ag layered feedstock (400 nm Cu at the top) at 300°C and 40 nm, fabricated nanowires are Ag-Cu heterostructures standing on pure Ag substrate.

Case (x): When using B-A layered feedstock (B at the top) and processing at temperature $T < T_{c,\mathrm{A}}$, one can fabricate nanowires of B-A heterostructure that stand on a substrate of A. In this case, B nanowires form first until all B atoms are consumed. Then, A nanowires grow by dislocation slip and push the formed B parts up. One example of this case is TMNM of Au-Ag layered feedstock (50 nm Au at the top) at 50°C and 250 nm, fabricated nanowires are Au-Ag heterostructures standing on pure Ag substrate.

Case (xi): When using A-B layered feedstock (A at the top) and processing at temperature $T < T_{c,\mathrm{A}}$ and $T > T_{c,\mathrm{B}}$, one can fabricate nanowires of B-A heterostructure that stand on a substrate of B. In this case, A nanowires form first until all A atoms are consumed. Then, B atoms diffuse through the interface between A nanowires and mold, and build up nanowire on top of A by epitaxial growth. During the growth of B, the dislocation dominated mechanism prevents A atoms from mixing with the atomic flux of B and forming AB mixture nanowires. To achieve this case requires the slower diffuser (B) to have lower T_c.

It is also worthy to note that in all the cases above, the structure of heterostructures can be precisely controlled. This ability of precise controlling is another important aspect for further applications. Besides the chemical sequence discussed above, for A-B/B-A heterostructure nanowires, the structure is thoroughly defined by the length of each layer. Consider TMNM with A-B layered feedstock (A at the top) for example, the final length of A part in nanowires (L_A) can be precisely

calculated with the deposition thickness of A layer in feedstock (t_A) and the porosity (a) of the mold upon volume conservation:

$$L_A = t_A/a$$

Considering the length of B part (L_B) is $L_B = L - L_A$, L_B as well as the location of A-B interface can also be precisely controlled by controlling the total length of nanowires. In a diffusion dominated mechanism, L can be controlled by controlling molding time and pressure (eq. S5). And in a dislocation slip mechanism, one can tune the loading pressure (sometimes also diameter) to tune the maximum length L_m (eq. S9) and apply a reasonable molding time to achieve $L = L_m$. For example, when using Ag-Cu layered feedstock (200 nm Ag at the top) to fabricate Ag-Cu heterostructure nanowires (case (viii)), typical 40 nm or 80 nm molds with porosity $a \sim 15\%$ result in ~1.3 µm Ag parts in fabricated nanowires. Under a molding condition of 80 nm, 600 s, and 1.6 GPa, the total length ~ 6 µm, and Ag-Cu interface is ~ 4.5 µm away from substrate. By reducing the loading pressure to 0.8 GPa, or changing the molding diameter to 40 nm, we can reduce the total length by half (~ 3 µm), and the Ag-Cu interface is tuned to ~ 1.5 µm away from the substrate.

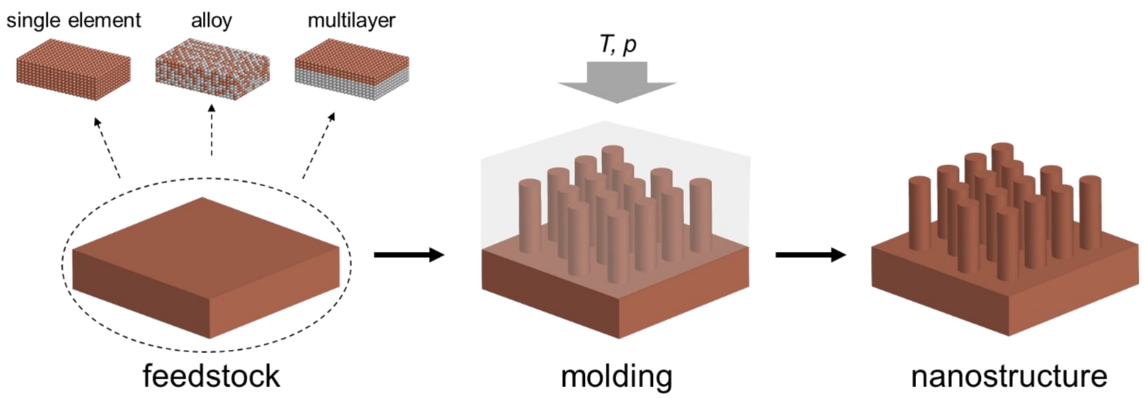

Fig S1.
Process of Thermomechanical Nanomolding (TMNM).

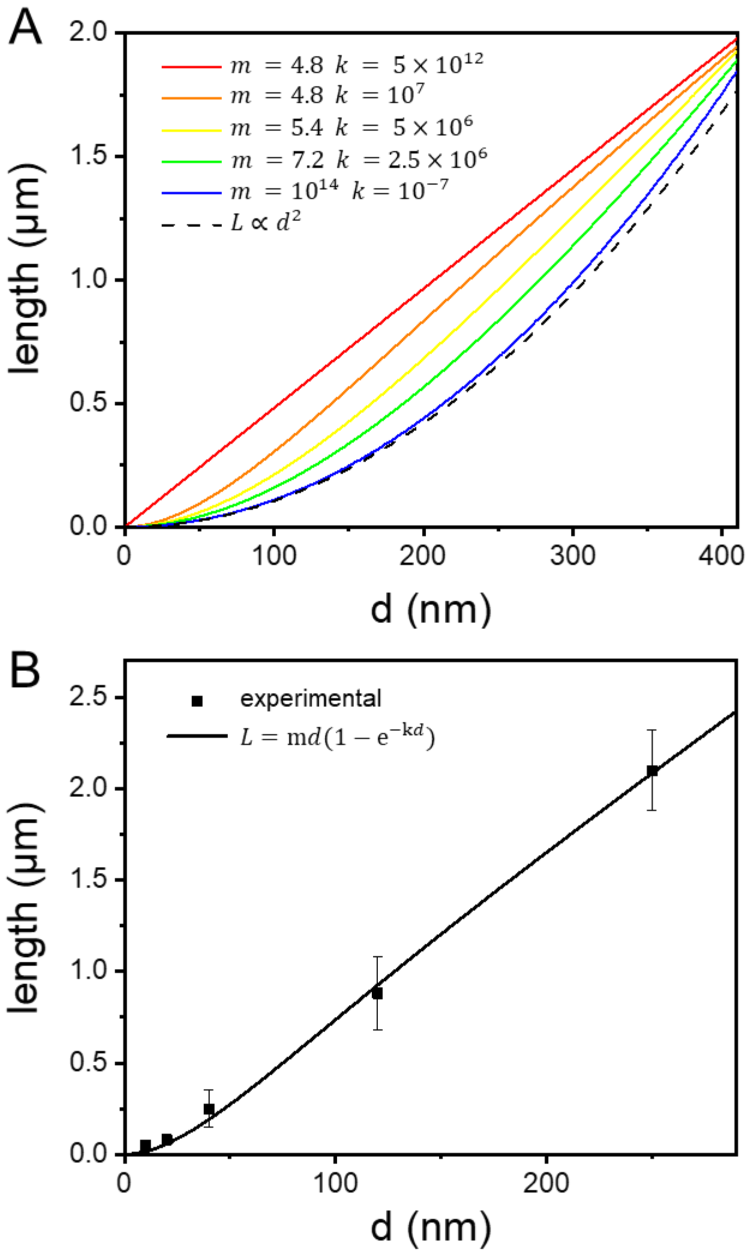

Fig S2.

Scaling behavior of dislocation mediated TMNM. **(A)** Numerical results of scaling behavior between L and d in dislocation slip mechanism (eq. 3 and eq. S9) in the format of $L = \mathrm{m}d(1 - \mathrm{e}^{-\mathrm{k}d})$. Here by tuning the constant m and k, the curve of $L(d)$ shifts between first and second order power function (see supplementary text for more detailed discussion). **(B)** Scaling results of Au at 0.4 T_m (262°C) fitted with eq. 3 (eq. S9) in the format of $L = \mathrm{m}d(1 - \mathrm{e}^{-\mathrm{k}d})$. Fitted curve is with $\mathrm{m} = 8.4$ and $\mathrm{k} = 2.1 \times 10^{7}$, resulting in R^2 of 0.9976.

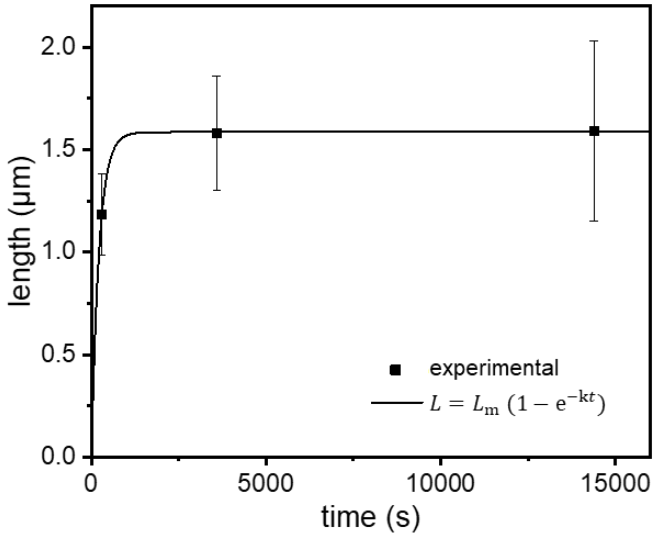

Fig S3.
Scaling behavior of L vs. t in dislocation slip mechanism. Superimposed data is collected with TMNM of Ag at 100°C, 250 nm and a loading pressure of 1.6 GPa. These data are fitted with eq. 3 (eq. S9) in format $L = L_{\mathrm{m}}(1 - \mathrm{e}^{-\mathrm{k}t})$, and result in $R^2 = 0.9996$.

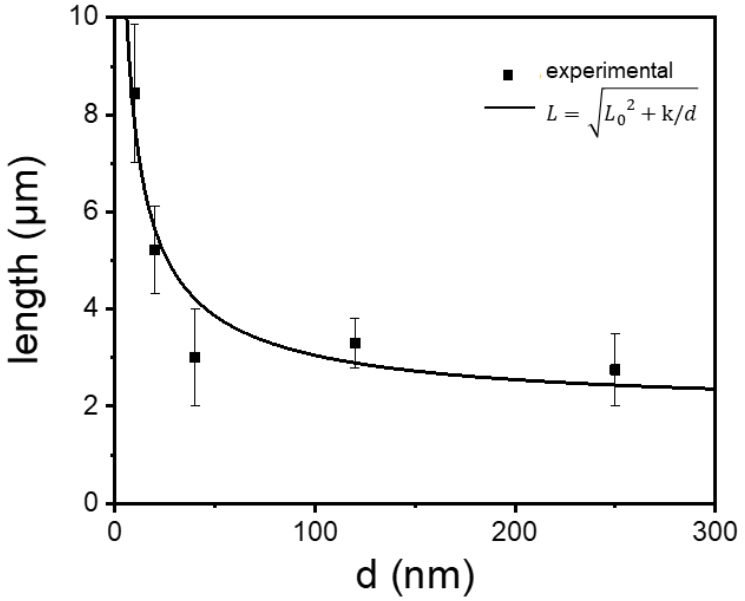

Fig S4.
Scaling behavior of L vs. d of Au in diffusion region (529°C, $0.6T_m$). A fitting curve with eq. 2 (eq. S5) shows good matching between experimental data and theory. $R^2 \sim 0.8945$.

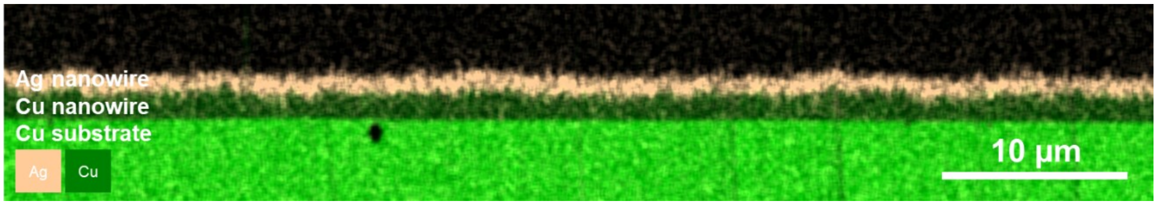

Fig S5.
Cross-section view of example heterostructured nanowire arrays fabricated by TMNM. These nanowires are composed by Cu and Ag separated by a sharp interface. Diameter of the nanowires is 40 nm.

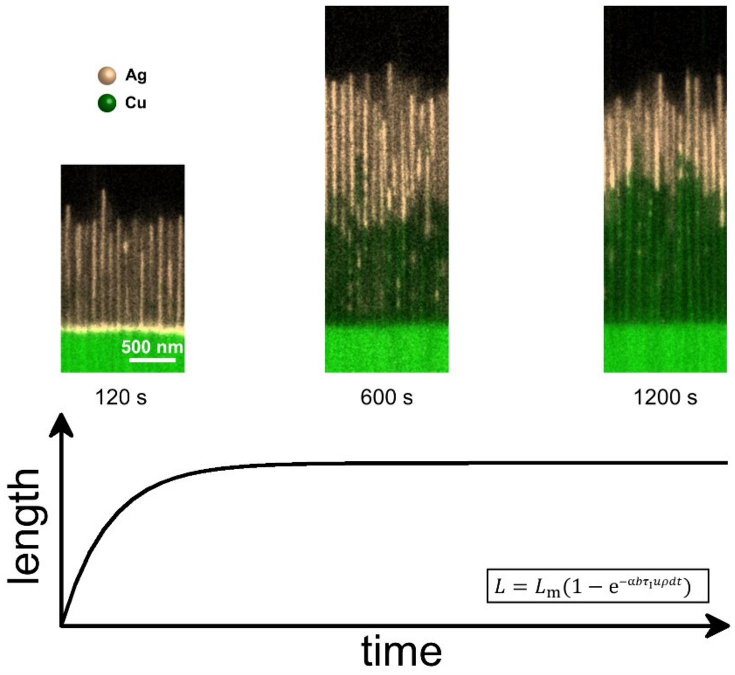

Fig S6.
Forming of heterostructure nanowires as in the case of Fig. 3A. When using Ag on the top facing the nanocavities and Cu at the bottom, Ag nanowires form first (as shown at 120 s) until all Ag atoms of the feedstock are consumed building up nanowires. Then, Cu nanowires grow up below those existed Ag nanowires by dislocation slip, and push the Ag nanowires up (as shown at 600 s and 1200 s). After formation of the Ag parts, the total length (L) (dark line) of these heterostructure nanowires is contributed by the growth of Cu parts and follows the scaling of L vs. t of the dislocation mechanism (eq. 3 and eq. S9). In this case, L stops increasing after the molding pressure gets a balance with the resistance of the mold-nanowire interface. The molding conditions are T = 300°C, d = 40 nm, and a loading pressure of 1.6 GPa.

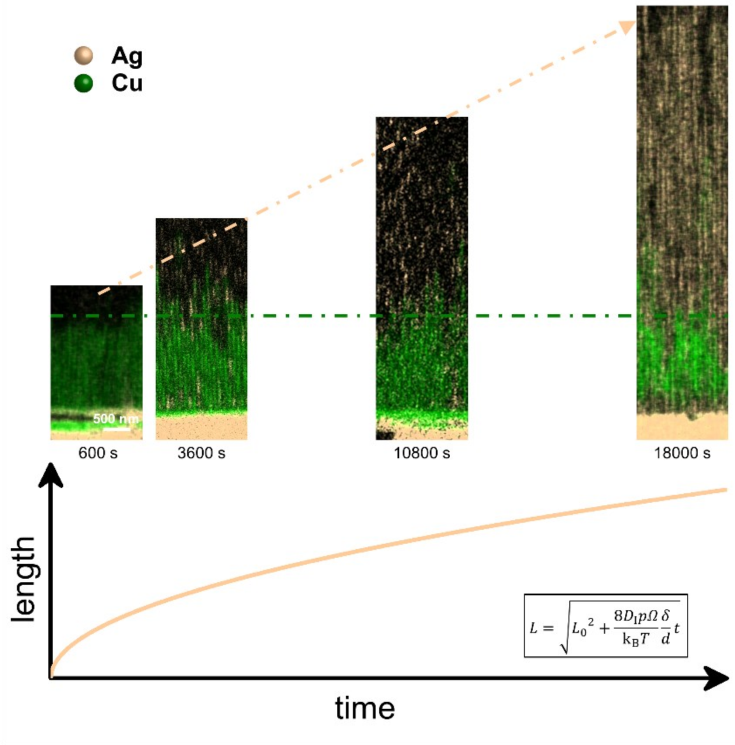

Fig S7.
Forming of heterostructure nanowires as in the case of Fig. 3B. When using Cu on the top facing the nanocavities and Ag at the bottom, Cu nanowires form first (as shown at 600 s) until all Cu atoms of the feedstock are consumed building up nanowires. Then, Ag nanowires grow up on the top of those existed Cu nanowires through interface diffusion (as shown at 3600 s, 10800 s, and 18000 s). During the growth of Ag parts, the total length of formed nanowires keeps increasing, while the length of Cu parts remains the same (marked by the green dash line). As the total length (L) is mainly contributed by the growth up of Ag through diffusion (light orange line), the scaling of L vs. t follows eq. 2 (eq. S5) and $L \propto \sqrt{t}$. The molding conditions are T = 300°C, d = 40 nm, and a loading pressure of 1.6 GPa.

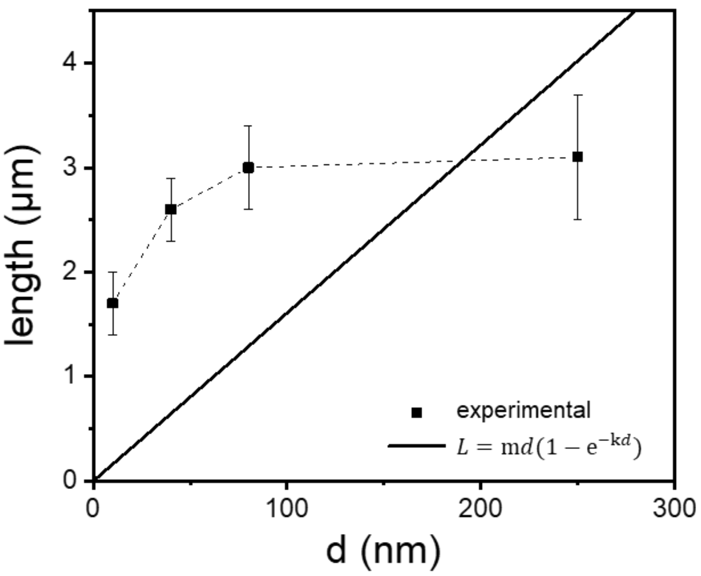

Fig S8.
Scaling results of L vs. d of Cu at 400°C (0.5 T_m). The molding length is increasing with molding diameter but not following the prediction of dislocation slip model. For smaller d, the molding length is longer than the scaling of eq. 3 (eq. S9), and showing a mixture of interface diffusion and dislocation slip mechanisms. This result is a sign for the mechanism transition from diffusion to dislocation.

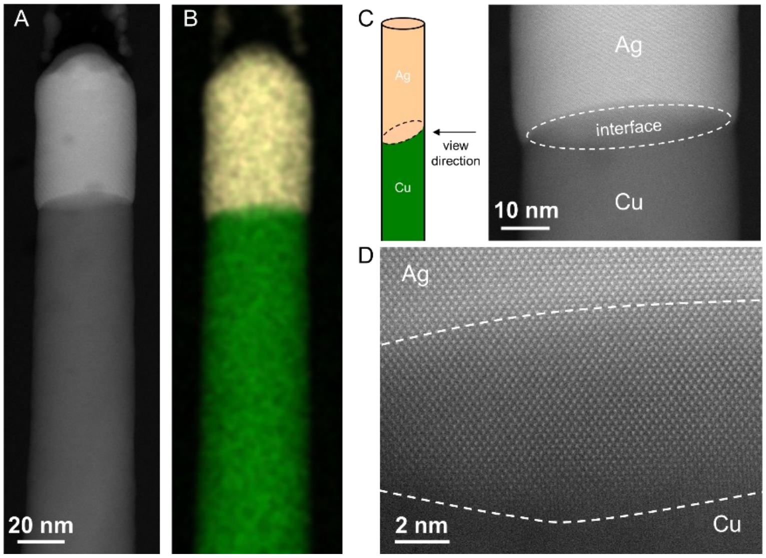

Fig S9.
Interface of molded heterostructure nanowire characterized by STEM. **(A)** STEM image of a typical Cu-Ag heterostructure nanowire fabricated by TMNM. The contrast is from the difference of z-factor between Ag (the top part) and Cu (the bottom part). **(B)** EDS mapping of the heterostructure nanowire. **(C)** When the Cu-Ag interface tilts from the cross-section, it projects to the view direction and forms an ellipse in TEM image. **(D)** High resolution STEM image of the interface region in (C), showing vanishing lattice of the single-crystal Ag part.

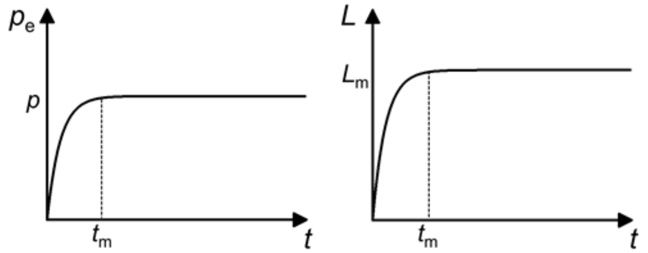

Fig S10.
p_e vs. t (left) and L vs. t (right) in dislocation dominated mechanism calculated from eq. S7 and S9. Here t_m marks the time when a nanowire approaches its maximum length L_m.

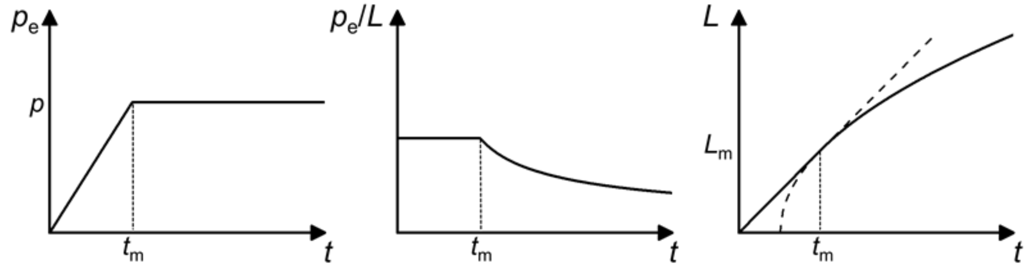

Fig S11.
p_e (left), p_e /L (middle) and L (right) vs. t in interface diffusion dominated mechanism calculated from eq. S12 and S13. Here t_m marks the time when p_e approaches the full value of the hydrostatic pressure (p). The dashed lines in the L vs. t plot are extrapolated from linear and square root equation (eq. S13).

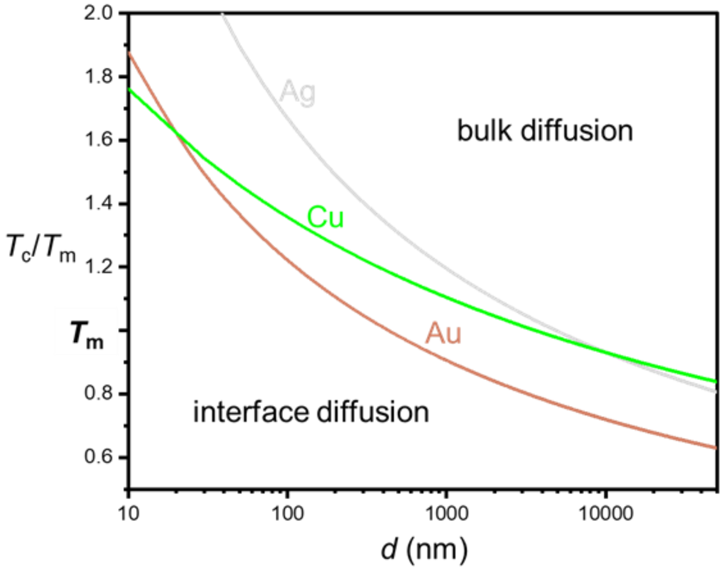

Fig S12.
Normalized mechanism transition temperature, T_c, from interface to bulk diffusion, as a function of molding diameter. For conditions above the $T_c(d)$ curve bulk diffusion is dominated and for conditions below, interface diffusion dominates. Data of diffusivities are collected from references (*28-30, 42, 44, 45*).

Table S1. Data superimposed in Fig. 2C.

material	$D_{I\,0}$ (m^2/s)	Q (eV)	ref.
In	3.7	0.81091	(*46*)
Sn	1.8×10^{-9}	0.46	(*41*)
Al	1.9×10^{-1}	1.28	(*47*)
Au	3.1×10^{-7}	0.88	(*30*)
Cu	1.2×10^{-6}	0.88	(*29*)
Ni	7×10^{-6}	1.1882	(*48*)
Fe	2×10^{-1}	2.78	(*49*)
Pt	2.2×10^{-1}	2.89	(*50*)
W	6.85×10^{1}	6.961	(*51*)
Sb	2.22×10^{1}	2.0424	(*52*)
Ge	7.8	2.9704	(*53*)
Si	4.23×10^{2}	4.73	(*54*)
InSb	5.3×10^{-4}	1.91	(*55*)
$AuIn_2$	1.2×10^{-7}	0.52	(*56*)
SnTe	5×10^{-2}	1.78	(*57*)
SiO2	1.378	4.74	(*58*)
Al_2O_3	4.6	5.877	(*59*)

REFERENCES AND NOTES

1. M. R. Begley, D. S. Gianola, T. R. Ray, Bridging functional nanocomposites to robust macroscale devices. *Science* **364**, eaav4299 (2019).

2. J. A. Liddle, G. M. Gallatin, Nanomanufacturing: A perspective. *ACS Nano* **10**, 2995–3014 (2016).

3. N. Lassaline, R. Brechbühler, S. J. W. Vonk, K. Ridderbeek, M. Spieser, S. Bisig, B. le Feber, F. T. Rabouw, D. J. Norris, Optical Fourier surfaces. *Nature* **582**, 506–510 (2020).

4. R. X. Yan, D. Gargas, P. D. Yang, Nanowire photonics. *Nat. Photonics* **3**, 569–576 (2009).

5. X. Feng, M. E. Tousley, M. G. Cowan, B. R. Wiesenauer, S. Nejati, Y. Choo, R. D. Noble, M. Elimelech, D. L. Gin, C. O. Osuji, Scalable fabrication of polymer membranes with vertically aligned 1 nm pores by magnetic field directed self-assembly. *ACS Nano* **8**, 11977–11986 (2014).

6. J. Kim, H. C. Lee, K. H. Kim, M. S. Hwang, J. S. Park, J. M. Lee, J. P. So, J. H. Choi, S. H. Kwon, C. J. Barrelet, H. G. Park, Photon-triggered nanowire transistors. *Nat. Nanotechnol.* **12**, 963–968 (2017).

7. W. H. Li, K. Ding, H. R. Tian, M. S. Yao, B. Nath, W. H. Deng, Y. Wang, G. Xu, Conductive metal-organic framework nanowire array electrodes for high-performance solid-state supercapacitors. *Adv. Funct. Mater.* **27**, 1702067 (2017).

8. K. Ding, J. Wang, Y. Zhou, H. Tian, L. Lu, R. Mazzarello, C. Jia, W. Zhang, F. Rao, E. Ma, Phase-change heterostructure enables ultralow noise and drift for memory operation. *Science* **366**, 210–215 (2019).

9. J. Padmanabhan, E. R. Kinser, M. A. Stalter, C. Duncan-Lewis, J. L. Balestrini, A. J. Sawyer, J. Schroers, T. R. Kyriakides, Engineering cellular response using nanopatterned bulk metallic glass. *ACS Nano* **8**, 4366–4375 (2014).

10. Z. Gao, A. Agarwal, A. D. Trigg, N. Singh, C. Fang, C.-H. Tung, Y. Fan, K. D. Buddharaju, J. Kong, Silicon nanowire arrays for label-free detection of DNA. *Anal. Chem.* **79**, 3291–3297 (2007).

11. T. Fujita, P. Guan, K. McKenna, X. Lang, A. Hirata, L. Zhang, T. Tokunaga, S. Arai, Y. Yamamoto, N. Tanaka, Y. Ishikawa, N. Asao, Y. Yamamoto, J. Erlebacher, M. Chen, Atomic origins of the high catalytic activity of nanoporous gold. *Nat. Mater.* **11**, 775–780 (2012).

12. W. Wang, F. Lv, B. Lei, S. Wan, M. Luo, S. Guo, Tuning nanowires and nanotubes for efficient fuel-cell electrocatalysis. *Adv. Mater.* **28**, 10117–10141 (2016).

13. S. Gazibegovic, D. Car, H. Zhang, S. C. Balk, J. A. Logan, M. W. A. de Moor, M. C. Cassidy, R. Schmits, D. Xu, G. Wang, P. Krogstrup, R. L. M. op het Veld, K. Zuo, Y. Vos, J. Shen, D. Bouman, B. Shojaei, D. Pennachio, J. S. Lee, P. J. van Veldhoven, S. Koelling, M. A. Verheijen, L. P. Kouwenhoven, C. J. Palmstrøm, E. P. A. M. Bakkers, Epitaxy of advanced nanowire quantum devices. *Nature* **548**, 434–438 (2017).

14. G. Kumar, H. X. Tang, J. Schroers, Nanomoulding with amorphous metals. *Nature* **457**, 868–872 (2009).

15. Y. C. Chou, K. Hillerich, J. Tersoff, M. C. Reuter, K. A. Dick, F. M. Ross, Atomic-scale variability and control of III-V nanowire growth kinetics. *Science* **343**, 281–284 (2014).

16. G. Zhang, M. Takiguchi, K. Tateno, T. Tawara, M. Notomi, H. Gotoh, Telecom-band lasing in single InP/InAs heterostructure nanowires at room temperature. *Sci. Adv.* **5**, eaat8896 (2019).

17. H. Winnischofer, T. C. R. Rocha, W. C. Nunes, L. M. Socolovsky, M. Knobel, D. Zanchet, Chemical synthesis and structural characterization of highly disordered Ni colloidal nanoparticles. *ACS Nano* **2**, 1313–1319 (2008).

18. C. B. Murray, C. R. Kagan, M. G. Bawendi, Synthesis and characterization of monodisperse nanocrystals and close-packed nanocrystal assemblies. *Annu. Rev. Mater. Sci.* **30**, 545–610 (2000).

19. Z. Liu, One-step fabrication of crystalline metal nanostructures by direct nanoimprinting below melting temperatures. *Nat. Commun.* **8**, 14910 (2017).

20. N. Liu, Y. Xie, G. Liu, S. Sohn, A. Raj, G. Han, B. Wu, J. J. Cha, Z. Liu, J. Schroers, General nanomolding of ordered phases. *Phys. Rev. Lett.* **124**, 036102 (2020).

21. Z. Liu, G. Han, S. Sohn, N. Liu, J. Schroers, Nanomolding of crystalline metals: The smaller the easier. *Phys. Rev. Lett.* **122**, 036101 (2019).

22. Z. Liu, Investigation of temperature and feature size effects on deformation of metals by superplastic nanomolding. *Phys. Rev. Lett.* **122**, 016101 (2019).

23. J. K. Hyun, S. Zhang, L. J. Lauhon, Nanowire heterostructures. *Annu. Rev. Mat. Res.* **43**, 451–479 (2013).

24. R. Agarwal, Heterointerfaces in semiconductor nanowires. *Small* **4**, 1872–1893 (2008).

25. S. Kral, C. Zeiner, M. Stöger-Pollach, E. Bertagnolli, M. I. den Hertog, M. Lopez-Haro, E. Robin, K. el Hajraoui, A. Lugstein, Abrupt Schottky junctions in Al/Ge nanowire heterostructures. *Nano Lett.* **15**, 4783–4787 (2015).

26. Y. Wu, J. Xiang, C. Yang, W. Lu, C. M. Lieber, Erratum: Single-crystal metallic nanowires and metal/semiconductor nanowire heterostructures. *Nature* **430**, 704–704 (2004).

27. L. Y. Chen, M. R. He, J. Shin, G. Richter, D. S. Gianola, Measuring surface dislocation nucleation in defect-scarce nanostructures. *Nat. Mater.* **14**, 707–713 (2015).

28. R. E. Hoffman, D. Turnbull, Lattice and grain boundary self-diffusion in silver. *J. Appl. Phys.* **22**, 634–639 (1951).

29. T. Surholt, C. Herzig, Grain boundary self-diffusion in Cu polycrystals of different purity. *Acta Mater.* **45**, 3817–3823 (1997).

30. D. Gupta, Grain-boundary self-diffusion in Au by Ar sputtering technique. *J. Appl. Phys.* **44**, 4455–4458 (1973).

31. M. E. Kassner, *Fundamentals of Creep in Metals and Alloys* (Butterworth-Heinemann, 2015).

32. L. Zhong, F. Sansoz, Y. He, C. Wang, Z. Zhang, S. X. Mao, Slip-activated surface creep with room-temperature super-elongation in metallic nanocrystals. *Nat. Mater.* **16**, 439–445 (2017).

33. T. Zhu, J. Li, Ultra-strength materials. *Prog. Mater. Sci.* **55**, 710–757 (2010).

34. G. Cheng, C. Miao, Q. Qin, J. Li, F. Xu, H. Haftbaradaran, E. C. Dickey, H. Gao, Y. Zhu, Large anelasticity and associated energy dissipation in single-crystalline nanowires. *Nat. Nanotechnol.* **10**, 687–691 (2015).

35. Q. Qin, S. Yin, G. Cheng, X. Li, T.-H. Chang, G. Richter, Y. Zhu, H. Gao, Recoverable plasticity in penta-twinned metallic nanowires governed by dislocation nucleation and retraction. *Nat. Commun.* **6**, 5983 (2015).

36. P. Guo, J. Xu, K. Gong, X. Shen, Y. Lu, Y. Qiu, J. Xu, Z. Zou, C. Wang, H. Yan, Y. Luo, A. Pan, H. Zhang, J. C. Ho, K. M. Yu, On-nanowire axial heterojunction design for high-performance photodetectors. *ACS Nano* **10**, 8474–8481 (2016).

37. B. M. Borg, K. A. Dick, B. Ganjipour, M. E. Pistol, L. E. Wernersson, C. Thelander, InAs/GaSb heterostructure nanowires for tunnel field-effect transistors. *Nano Lett.* **10**, 4080–4085 (2010).

38. X. M. Zhang, M. Y. Lu, Y. Zhang, L. J. Chen, Z. L. Wang, Fabrication of a high-brightness blue-light-emitting diode using a ZnO-nanowire array grown on p-GaN thin film. *Adv. Mater.* **21**, 2767–2770 (2009).

39. M. Huang, “Modelling plastic deformation of metals using irreversible thermodynamics,” thesis, Shanghai Jiaotong University, Shanghai (2008).

40. R. W. Balluffi, J. M. Bkakely, Special aspects of diffusion in thin films. *Thin Solid Films* **25**, 363–392 (1975).

41. P. Sun, M. Ohring, Tracer self-diffusion and electromigration in thin tin films. *J. Appl. Phys.* **47**, 478–485 (1976).

42. S. M. Makin, A. H. Rowe, A. D. Leclaire, Self-diffusion in gold. *Proc. Phys. Soc. B* **70**, 545–552 (1957).

43. O. Kubaschewski, The diffusion rates of some metals in copper, silver, and gold. *Trans. Faraday Soc.* **46**, 713–722 (1950).

44. C. T. Tomizuka, E. Sonder, Self-diffusion in silver. *Phys. Rev.* **103**, 1182–1184 (1956).

45. J. Steigman, W. Shockley, F. C. Nix, The self-diffusion of copper. *Phys. Rev.* **56**, 13–21 (1939).

46. J. E. Dickey, Self-diffusion in indium. *Acta Metall.* **7**, 350–353 (1959).

47. J. Burke, T. R. Ramachandran, Self-diffusion in aluminum at low temperatures. *Metall. Mater. Trans. B* **3**, 147–155 (1972).

48. A. R. Wazzan, Lattice and grain boundary self-diffusion in nickel. *J. Appl. Phys.* **36**, 3596–3599 (1965).

49. D. Graham, D. H. Tomlin, Self-diffusion in iron. *Philos. Mag.* **8**, 1581–1585 (1963).

50. F. Cattaneo, F. Grasso, E. Germagnoli, Self-diffusion in platinum. *Philos. Mag.* **7**, 1373–1383 (1962).

51. J. N. Mundy, S. J. Rothman, N. Q. Lam, H. A. Hoff, L. J. Nowicki, Self-diffusion in tungsten. *Phys. Rev. B* **18**, 6566–6575 (1978).

52. H. B. Huntington, P. B. Ghate, J. H. Rosolowski, Self-diffusion in antimony. *J. Appl. Phys.* **35**, 3027–3032 (1964).

53. H. Letaw Jr., W. M. Portnoy, L. Slifkin, Self-diffusion in germanium. *Phys. Rev.* **102**, 636–639 (1956).

54. T. Südkamp, H. Bracht, Self-diffusion in crystalline silicon: A single diffusion activation enthalpy down to755°C. *Phys. Rev. B* **94**, 125208 (2016).

55. A. Rastogi, K. V. Reddy, Self-diffusion in indium antimonide. *J. Appl. Phys.* **75**, 4984–4989 (1994).

56. Y. Hasumi, Lateral diffusion of In and formation of $AuIn_2$ in Au-In thin films. *J. Appl. Phys.* **58**, 3081–3086 (1985).

57. H. Scherrer, G. Pineau, S. Scherrer, Diffusion of ^{117}Sn in tin telluride. *Phys. Lett. A* **75**, 118–120 (1979).

58. D. Tsoukalas, C. Tsamis, P. Normand, Diffusivity measurements of silicon in silicon dioxide layers using isotopically pure material. *J. Appl. Phys.* **89**, 7809–7813 (2001).

59. A. H. Heuer, Oxygen and aluminum diffusion in α-Al_2O_3: How much do we really understand? *J. Eur. Ceram. Soc.* **28**, 1495–1507 (2008).
